# Sensitive Detection of Thirteen Bacterial Vaginosis-Associated Agents Using Multiplex Polymerase Chain Reaction

**DOI:** 10.1155/2015/645853

**Published:** 2015-05-20

**Authors:** Natália Malaguti, Larissa Danielle Bahls, Nelson Shozo Uchimura, Fabrícia Gimenes, Marcia Edilaine Lopes Consolaro

**Affiliations:** ^1^Clinical Cytology and STD Laboratory, Department of Clinical Analysis and Biomedicine, State University of Maringá, 87020900 Maringá, PR, Brazil; ^2^Department of Medicine, State University of Maringá, 87020900 Maringá, PR, Brazil

## Abstract

Bacterial vaginosis (BV) is characterized by a polymicrobial proliferation of anaerobic bacteria and depletion of lactobacilli, which are components of natural vaginal microbiota. Currently, there are limited conventional methods for BV diagnosis, and these methods are time-consuming, expensive, and rarely allow for the detection of more than one agent simultaneously. Therefore, we conceived and validated a multiplex polymerase chain reaction (M-PCR) assay for the simultaneous screening of thirteen bacterial vaginosis-associated agents (BV-AAs) related to symptomatic BV: *Gardnerella vaginalis*, *Mobiluncus curtisii*, *Mobiluncus mulieris*, *Bacteroides fragilis*, *Mycoplasma hominis*, *Atopobium vaginae*, *Ureaplasma urealyticum*, *Megasphaera* type I, Clostridia-like bacteria vaginosis-associated bacteria (BVABs) 1, 2, and 3, *Sneathia sanguinegens*, and *Mycoplasma genitalium*. The overall validation parameters of M-PCR compared to single PCR (sPCR) were extremely high, including agreement of 99.1% and sensitivity, specificity, and positive predictive values of 100.0%, negative predictive value of 97.0%, accuracy of 99.3%, and agreement with Nugent results of 100.0%. The prevalence of BV-AAs was very high (72.6%), and simultaneous agents were detected in 53.0%, which demonstrates the effectiveness of the M-PCR assay. Therefore, the M-PCR assay has great potential to impact BV diagnostic methods in vaginal samples and diminish associated complications in the near future.

## 1. Introduction

Bacterial vaginosis (BV) is the most prevalent lower genital tract infection in women of reproductive age worldwide [1]. Women with BV typically report symptoms that include a thin vaginal discharge and a fishy malodor [2]. However, a substantial portion of affected women is asymptomatic [3]. The etiology of BV is not completely understood. No single etiological agent is the known cause of BV, and the syndrome is considered an ecological disorder of the vaginal microbiota. BV is characterized by a reduction of lactic acid-producing bacteria (mainly* Lactobacillus* spp.) and an increase in the number and diversity of facultative and strictly anaerobic bacteria [4–6]. BV is an independent risk factor for adverse outcomes, including pelvic inflammatory disease (PID) [7, 8] and subsequent infertility [9], increased risk of preterm labor and delivery [9, 10], amniotic fluid infections [11], chorioamnionitis [12], low birth weight [13], endometritis [14], cervicitis [15], and an increased risk of acquiring sexually transmitted infections (STIs) and HIV [5, 16]. Therefore, the diagnosis of BV is essential, especially in pregnant females, as early as possible to prevent complications [17]. The precise pathophysiology and epidemiology of BV and the optimal medical management of the condition are far from clear. Much of this lack of understanding is a direct result of the difficulty in establishing a diagnostic standard for this syndrome [18].

BV is often diagnosed clinically based on the criteria described by Amsel et al. [19], wherein three of the following four signs must be evident: vaginal fluid pH greater than 4.5; homogeneous vaginal discharge on examination; detection of a fishy odor upon addition of 10% potassium hydroxide to vaginal fluid; and the presence of significant clue cells (>20%). Another method that is widely used for BV diagnosis is based on grading or scoring the microbiota in Gram-stained smears of vaginal fluid (Nugent scoring) [20]. BV diagnosis in research and laboratory settings depends on traditional methods, such as culture and Gram-staining vaginal smears [5, 21, 22]. Conventional microbiological approaches have only limited utility in evaluating patients for BV. The hallmark of the condition is a complex perturbation of the normal vaginal microbiota, and culture-based identification of single “marker” organisms lacks sensitivity and specificity [23]. Additionally, many of the key organisms that are associated with BV are obligate anaerobes that are either difficult to recover or unrecoverable using conventional culture methods, which makes a true evaluation of vaginal microbiota using culture impossible [6].

The true extent of the microbial diversity in BV was indicated only with the advent of recent cultivation-independent molecular-based approaches, such as polymerase chain reaction (PCR), multiplex-PCR (M-PCR), real-time PCR, taxon-directed PCR, broad-range bacterial 16S rDNA PCR, and fluorescence in situ hybridization (FISH) [10, 23–30]. These techniques demonstrated the association of several novel bacteria with BV [29]. Actually, common bacterial vaginosis-associated agents (BV-AAs) include* Gardnerella vaginalis*,* Mobiluncus *spp.,* Mycoplasma hominis*,* Atopobium vaginae,* and* Bacteroides fragilis*, wherein the presence of* G. vaginalis* and* A. vaginae* together in high copy numbers has a high sensitivity (95%) and specificity (99%) for the prediction of BV [10, 24, 31]. BV-AAs include Clostridia-like bacteria vaginosis-associated bacteria (BVABs) 1, 2, and 3,* Sneathia *spp.,* Megasphaera *type I,* Ureaplasma urealyticum,* and* Mycoplasma genitalium* [10, 32–36]. Fredricks et al. [32] demonstrated that the presence of BVAB 2 or* Megasphaera* type I has a sensitivity of 100% and specificity of 91.3% for the prediction of BV. Ling et al. [10] showed that* Megasphaera* type I and* Sneathia *spp. were detected at a higher prevalence and higher relative abundance in women with BV.

We report a validated M-PCR diagnostic assay to simultaneous screen for thirteen BV-AAs related to BV:* Gardnerella vaginalis, Mobiluncus curtisii, Mobiluncus mulieris, Bacteroides fragilis, Mycoplasma hominis, Atopobium vaginae, Ureaplasma urealyticum, Megasphaera* type I, BVAB 1, BVAB 2, BVAB 3,* Sneathia sanguinegens,* and* Mycoplasma genitalium*. We believe that M-PCR will potentially impact the diagnostics of BV and diminish the associated complications in the near future.

## 2. Materials and Methods

### 2.1. Study Population and Sample Collection

From February 2013 to March 2014, 223 unselected women who attended the Clinical and Research Laboratory (LEPAC) of the State University of Maringá (UEM)/Brazil for regular cervical cancer screening (Pap) upon doctor referral who agreed to participate and fulfilled the inclusion criteria were enrolled in the study. Accordingly, all women were nonpregnant, of reproductive age (range, 15–54 years; mean, 34 years; median, 32 years), had not been using antimicrobials (oral or topical) within the previous 4 weeks, and had not been using an intrauterine device or contraceptives delivered directly to the vaginal mucosa. The Committee for Ethics in Research Involving Humans at the State University of Maringá (UEM)/Paraná, Brazil, approved this study (number 085/2011 and number 104/2012), and each woman involved signed a consent form.

Gram stains of vaginal fluid were not performed routinely on subjects from the LEPAC, in which the patients were recruited. This analysis is performed only upon medical request. Therefore, we included initially 45 samples from women with previous BV diagnosis using Nugent criteria (maximum 15 days prior to study collection), who had not yet received treatment, to standardize the M-PCR method to simultaneous screen for thirteen BV-AAs. Subsequently, the remaining 178 samples were included to validate the assay, and a total of 223 samples from an equal number of women were included.

Vaginal samples for molecular analysis were collected using Ayre's spatula prior to collection of Pap and Gram smears. Samples were transferred to tubes containing 1.0 mL of sterile 0.9% NaCl solution and immediately stored at −80°C until DNA extraction.

### 2.2. Multiplex-PCR for the Detection of Thirteen BV-AAs

#### 2.2.1. Genomic DNA Extraction

An AxyPrep Body Fluid Viral DNA/RNA Miniprep Kit (Axygen, CA, USA) was used according to the manufacturer's instructions. The quality and quantity of purified DNA were measured using spectrophotometry (NanoDrop 2000 Spectrophotometer, Thermo Scientific, Wilmington, USA).

#### 2.2.2. Design of Primers

The thirteen primers were selected from published papers [24, 37–44]. Specificity was checked against all sequences in GenBank using SeqSearch, and primers were aligned by using the Clustal X program (version 1.81, NCBI, Bethesda, MD). Subsequently, all primers were evaluated by performing a Basic Local Alignment Search Tool (BLAST) analysis against the sequences in the NCBI database. The primers were approved when there are no mistakes in their critical regions (e.g., no mismatch at the 3′ end of a primer). All selected primers were designed to have similar physical characteristics to allow simultaneous amplification in a multiplex reaction without the loss of sensitivity and easy separation using gel electrophoresis as follows: melting temperatures (55°C to 65°C), length (18- to 26-base pair-bp), and amplicon sizes (80 to 842 bp) (Table 1).

To assess the specificity of the primers, all primers were also tested in either a sPCR or M-PCR reactions with different samples. No cross-reactivity among the all primers was observed upon amplification of clinical samples that tested positive for any of the 13 bacteria by routine diagnostic analysis.

#### 2.2.3. M-PCR Conditions

Different parameters (magnesium chloride-MgCl_2_ and primers concentration, annealing and extension temperatures, and number of cycles) were tested in different combinations. Different annealing temperatures for the primers were found, and we split our analysis into three M-PCR assays.

M-PCR assay 1 was standardized to detect six BV-AAs:* G. vaginalis*,* M. curtisii*,* B. fragilis*,* M. hominis*,* U. urealyticum,* and *M*. type I. Assay 2 detected three BV-AAs:* A. vaginae*, BVAB 1, and BVAB 2. Assay 3 detected four BV-AAs:* M. mulieris*, BVAB 3,* S. sanguinegens,* and* M. genitalium*. The annealing temperatures for each assay were 55°C, 62°C, and 63°C, respectively.

The optimized protocol for each assay was a mixture of 25 *μ*L containing 2.5 mM of each of the deoxynucleotide triphosphates (dNTP), 0.6 mM of MgCl_2_, 25 mM of each primer, 5 *μ*L of extracted DNA (50 ng of total sample), and 1 U of* Platinum Taq* DNA polymerase (Invitrogen, CA, USA). The PCR conditions were comprised of thirty-five amplification cycles of denaturation for 10 min at 94°C, annealing for 1 min with variable temperature depending on the assay (55°C or 62°C or 63°C), extension for 1 min at 72°C, and final extension for 10 min at 72°C (Thermal cycler, Biosystem, CA, USA). M-PCR products were electrophoresed in 8% polyacrylamide gel stained with 1 *μ*g/mL ethidium bromide.

Positive controls for all studied BV-AAs were derived from positive clinical samples that were detected using reference methods, including culture and/or single PCR (sPCR). sPCR was also performed for the thirteen bacteria in all samples studied and positive controls using the same primers as the M-PCR for standardization and validation. sPCR (gold standard) is generally more sensitive than M-PCR, and cross-reactivity, which can occur during M-PCR, is avoided [45]. However, coamplification of the human *β*-globin gene using specific primers GH20/PC04 was performed in all clinical samples and controls as an internal control for amplification to ensure that amplifiable DNA was successfully extracted from the samples and monitored for PCR inhibitors under the same conditions as the M-PCR or sPCR reactions [46].

### 2.3. sPCR

sPCR was performed using the same primers as those used in the M-PCR and the assay consisted of 15 *μ*L containing 2.5 mM of each dNTP, 0.6 mM of MgCl_2_, 25 mM of each primer, 5 *μ*L of extracted DNA (50 ng of total sample), and 1 U of Taq DNA polymerase (Invitrogen, CA, USA). The PCR conditions were comprised of thirty-five amplification cycles of denaturation for 10 min at 94°C, annealing for 1 min at variable temperatures (depending on the BV-AAs temperature used in assay of M-PCR, being 55°C, 62°C, or 63°C), extension for 1 min at 72°C, and final extension for 10 min at 72°C (Thermal cycler, Biosystem, CA, USA). The amplification fragments were electrophoresed in 8% polyacrylamide gel stained with 1 *μ*g/mL ethidium bromide.

### 2.4. *β*-Globin PCR

To assess inhibition, sample adequacy, and integrity, each subject's extracted DNA was subjected to a coamplification of the human *β*-globin gene using primers GH20 (5′-GAAGAGCCAAGGACAGGTAC-3′) and PC04 (5′-CAACTTCATCCACGTTCACC-3′), under the same conditions as M-PCR or sPCR.

Two types of controls were also included in each reaction series (M-PCR, sPCR, and *β-globin *PCR), a “no-DNA” (negative control) and “HPV-positive DNA” (positive control).

### 2.5. Statistical Analysis

Statistical analysis was performed using Open Source Epidemiologic Statistics for Public Health/OpenEpi, Version 2.3.1. All variables were expressed as absolute and relative frequencies.

## 3. Results

### 3.1. M-PCR Assay Overall Performance

The M-PCR assay clearly distinguished and identified all thirteen BV-AAs in vaginal samples, whether only one (1 bacterium) or simultaneous bacteria (2 or more) were present, and false-positive results were not detected. Final results were regarded as true positives if the sPCR was also positive (gold standard).

**Figure 1:**
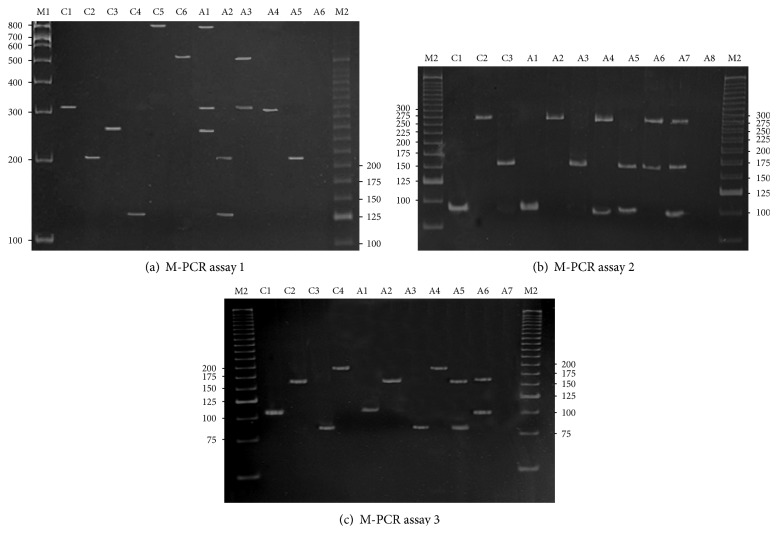
*Electrophoretic analysis of the amplified fragments by using M-PCR in 8*%* polyacrylamide gels*. Positive control (C). (a)* M-PCR assay 1*: C1:* Gardnerella vaginalis* (330 base pairs-bp); C2:* Megasphaera* type I (211 pb); C3:* Mycoplasma hominis *(270 pb); C4:* Mobiluncus curtisii *(130 pb); C5:* Bacteroides fragilis *(842 pb); C6:* Ureaplasma urealyticum* (541 pb); A1: positive sample for* B. fragilis*,* G. vaginalis* and* M. hominis*; A2: positive sample for *M*. type I and* M. curtisii*; A3: positive sample for* U. urealyticum* and* G. vaginalis*; A4: positive sample for* G. vaginalis*; A5: positive sample for *M*. type I (211 pb); A6: negative control. (b)* M-PCR assay 2*: C1: BVAB 1 (90 bp); C2: BVAB 2 (260 pb); C3:* Atopobium vaginae *(155 pb); A1: positive sample for BVAB 1; A2: positive sample for BVAB 2; A3: positive sample for* A. vaginae*; A4: positive sample for BVAB 2 and BVAB 1; A5: positive sample for* A. vaginae* and BVAB 1; A6: positive sample for BVAB 2 and* A. vaginae*; A7: positive sample of BVAB 2,* A. vaginae* and BVAB 1; A8: negative control. (c)* M-PCR assay 3*: C1:* Sneathia sanguinegens *(102 bp); C2: BVAB 3 (160 pb); C3:* Mobiluncus mulieris *(80 pb); C4:* Mycoplasma genitalium *(193 pb); A1: positive sample for* S. sanguinegens *(102 bp); A2: positive sample for BVAB 3; A3: positive sample of* Mobiluncus mulieris*; A4: positive sample of* M. genitalium*; A5: positive sample of BVAB 3 and* M. mulieris*; A6: positive sample for BVAB 3 and* S. sanguinegens*; A7: negative control. Lanes M1: molecular weight marker (100 bp); M2: molecular weight marker (25 bp).

The overall agreement of M-PCR results with sPCR was 99.1%, and the validation parameters were as follows: 100.0% sensitivity, specificity, and positive predictive value, 97.0% negative predictive value, and 99.3% accuracy. Individual analyses revealed that* G. vaginalis*,* Megasphaera *type I, BVAB 1,* U*.* urealyticum*, BVAB 3,* M. curtisii*,* A. vaginae, S. sanguinegens*,* M. mulieris*,* B. fragilis, *and* M. genitalium* showed values of 100.0% for all parameters of M-PCR. M-PCR showed 100.0% specificity and positive predictive values and 98.0% accuracy for both BVAB 2 and* M. hominis* agents. These agents differed in sensitivity (80.0% and 88.8%, resp.) and negative predictive value (97.8% and 97.6%, resp.) (Table 2).  Figure 1 shows the electrophoretic analyses of the amplified fragments using M-PCR in 8% polyacrylamide gel of positive samples for different BV-AAs.

### 3.2. M-PCR Assay Performance in Initial BV Positive Samples

The agreement of M-PCR with Nugent results was 100.0% because all 45 samples from women with previous BV diagnosis (Nugent criteria; score used: inclusion of abnormal and intermediate groups as positive BV diagnosis) initially included for M-PCR standardization showed BV-AAs.

In these 45 samples, 20 (44.5%) had only one BV-AAs; 10 (22.2%) had 2 simultaneous BV-AAs; 6 (13.3%) had 3 simultaneous BV-AAs; 6 (13.3%) had 4 simultaneous BV-AAs; 2 (4.4%) had 5 simultaneous BV-AAs; and 1 (2.2%) had 6 simultaneous BV-AAs. The most common BV-AAs detected as a single agent or simultaneously in these samples were* Megasphaera *type I (*n* = 21, 46.7%), followed by* G. vaginalis* (*n* = 14, 31.1%),* M. curtisii,* and BVAB 2 (*n* = 12, 26.7% each).* Bacteroides fragilis *was not detected.  Table 3 shows the M-PCR results for BV-AAs as single agent or simultaneously (two or more agents) in the 45 initially analyzed samples.

### 3.3. BV-AA Positivity in All Samples Analyzed

**Figure 2:**
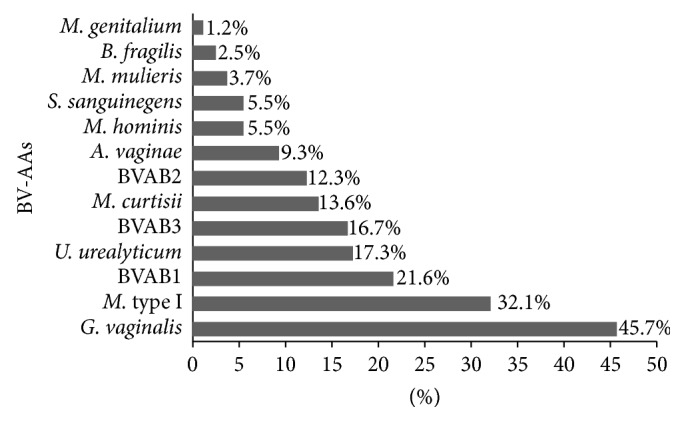
Frequency of thirteen major bacterial vaginosis-associated agents (BV-AAs) in all 223 samples studied as single agents or simultaneous detection.

BV-AAs were detected in 162 of the 223 samples (72.6%) as a single agent or simultaneously. The most frequent BV-AA was* G. vaginalis* (*n* = 74), which was detected in 45.7% of the positive samples, followed by *M*. type I (*n* = 52; 32.1%), BVAB 1 (*n* = 35; 21.6%),* U*.* urealyticum *(*n* = 28; 17.3%), BVAB 3 (*n* = 27; 16.7%),* M. curtisii* (*n* = 22; 13.6%), BVAB 2 (*n* = 20; 12.3%),* A. vaginae* (*n* = 15; 9.3%),* M. hominis *and* S. sanguinegens *(*n* = 9; 5.5%, each),* M. mulieris* (*n* = 6; 3.7%),* B. fragilis *(*n* = 4; 2.5%), and* M. genitalium* (*n* = 2; 1.2%) (Figure 2).

### 3.4. BV-AA Positivity as a Single Agent

Only one BV-AA was detected in 76 of the 162 positive samples (46.9%), which represented 34.1% of the total samples studied. The most frequent agent in these cases was* G. vaginalis* (*n* = 31; 40.8%), followed by *M*. type I (*n* = 14; 18.4%),* U*.* urealyticum *(*n* = 11; 14.5%),* M. curtisii* (*n* = 6; 7.9), and* M. hominis* (*n* = 4; 5.3%).

### 3.5. Simultaneous BV-AA Detection

Two or more BV-AAs were detected simultaneously in the remaining 86 BV-AA-positive samples (53.1%), which represented 38.6% of the total samples studied. Fifty of these samples (30.9%) showed two simultaneous BV-AAs, which represent 58.1% of all samples with detected simultaneous BV-AAs. The most frequent associations included* G. vaginalis *+ BVAB 1 (*n* = 7; 14.0%),* U*.* urealyticum *+* G. vaginalis *(*n* = 5; 10.0%),* M. *type I + BVAB 1, and *M*. type I +* G. vaginalis* (*n* = 4; 8.0%, each).

Three simultaneous BV-AAs were detected in 22 samples, which represented 13.6% of BV-AA-positive samples, 25.6% of total samples with simultaneous BV-AAs and 9.9% of the total samples studied. The most frequent associations were* A. vaginae *+ BVAB 2 + BVAB 3 and* G. vaginalis + M. curtisii* +* M. *type I (*n* = 2; 9.0%, each).

Four simultaneous BV-AAs were detected in 11 samples, which represented 6.8% of BV-AA-positive samples, 12.8% of the total samples with simultaneous BV-AAs and 4.9% of the total samples studied. The most frequent association was* U*.* urealyticum *+* G. vaginalis + M. *type I + BVAB 1 (*n* = 2; 18.2%).

Five simultaneous BV-AAs were detected in 2 samples, which represented 1.2% of positive BV-AA samples, 2.3% of total samples with simultaneous BV-AAs and 0.9% of the total samples studied. The associations were* M. curtisii* +* M.* type I + BVAB 3 +* M. mulieris *+* S. sanguinegens* and* U. urealyticum *+* G. vaginalis *+* A. vaginae *+ BVAB 1 + BVAB 2. Six simultaneous BV-AAs were detected in 1 sample, which represented 0.6% of positive BV-AA samples, 1.2% of total samples with simultaneous BV-AAs, and 0.4% of the total samples studied. The association was* G. vaginalis *+* M. curtisii *+* M.* type I +* A. vaginae *+ BVAB 2 +* M. mulieris*.

## 4. Discussion

To our knowledge, this is the first study to simultaneously screen for thirteen of the BV-AAs that are most related to BV in vaginal samples using M-PCR in Brazil and Latin America. Our study did not aim to identify or evaluate BV-AAs alone or in combination as markers of BV, but we sought to standardize and validate an M-PCR assay for the screening of populations in which the complications of BV may be more severe. The overall agreement of M-PCR with sPCR was elevated (99.1%), and other validation parameters, including sensitivity, specificity, positive and negative predictive value, and accuracy, were also excellent (ranging from 99.3% to 100%). M-PCR also showed excellent values for all parameters for the individual identification of BV-AAs, and M-PCR detected 86 cervical samples (53.0%) with two or more BV-AAs simultaneously. The agreement of M-PCR results with the Nugent method was 100.0%. Importantly, in the three M-PCR assays were used primers published in other studies without modification for all bacteria, but previously checked against all sequences in GenBank and evaluated by performing a BLAST analysis.

The application of this panel of 13 BV-AAs-targeted PCR assay to vaginal samples serves several purposes. First, these data help establish the bacterial compositions of the human cervix and vagina in subjects with and without BV and validate our earlier findings in smaller group BV-positive subjects using the Nugent method (*n* = 45). Second, the recent use of molecular microbial detection methods in well-characterized subjects established that a large portion of the vaginal microbiota in subjects with BV is derived from bacteria that appear to be novel and uncultivated [6, 27, 34]. To overcome this limitation, we developed a highly sensitive M-PCR assay targeting particular bacterial species that were previously detected in other molecular studies. Our approach will clearly not detect new species, but it is helpful in determining the true frequencies of key vaginal bacteria, which is a critical first step in understanding how vaginal bacteria interact with each other and the human host. Finally, rapid PCR assays may allow the microbiological diagnosis of BV in clinics [34] and clinical laboratories. The M-PCR assay is very sensitive assay that simplifies workflow and reduces costs and time, which allows for its use in routine diagnostic laboratories with basic molecular facilities [47–50]. Furthermore, application of the M-PCR assay will potentiate the diagnostics of BV-AAs because thirteen agents can be detected independently of the clinical status of women, wherein many of these BV-AAs are difficult to identify using conventional methods. We detected BV-AAs as single or simultaneous agents in 162 women from a total of 223. Therefore, this M-PCR assay has great potential for application in screening for BV-AAs in both pregnant and nonpregnant women as early as possible to prevent complications, such as PID [7, 8] and subsequent infertility [9], increased risk of preterm labor and delivery [9, 10], amniotic fluid infections [11], chorioamnionitis [12], low birth weight [13], endometritis [14], cervicitis [15], and increased susceptibility to infection with various pathogens, such as* Neisseria gonorrhoeae, Chlamydia trachomatis, Trichomonas vaginalis,* Herpes Simplex type-2 (HSV-2), and HIV [5, 16]. Below, we discuss only the frequency of the detected BV-AAs, recalling that our study did not aim to find or evaluate BV-AAs alone or in combination as markers of BV but to standardize and validate the use of the M-PCR assay.

The initial 45 samples were positive BV using the Nugent method, and the following BV-AAs were detected as a single agent or simultaneously:* Megasphaera *type I (46.7%), followed by* G. vaginalis* (31.1%),* M. curtisii,* and BVAB 2 (26.7% each). When all samples were studied together, the most prevalent BV-AAs detected were* G. vaginalis* (45.7%), followed by *M*. type I (32.1%), BVAB 1 (21.6%),* U. urealyticum* (17.3), BVAB 3 (16.7%), and* M. curtisii* (13.6%). Therefore, BV-AAs were detected more often individually in both samples, with the exception of* U. urealyticum*.

Cultivation methods failed to unequivocally identify a specific bacterial pathogen or unique pathogenic community in subjects with BV. Therefore, it had been hypothesized that several of the uncultivated bacteria associated with BV that were detected using PCR would be more reliable indicators of BV than the cultivated bacteria previously linked to this condition [34]. Our study obtained elevated values of validation parameters and detected that 53.1% of BV-AA-positive samples included two or more BV-AAs simultaneously: 30.9% showed two simultaneous bacteria, 25.6% three, 12.8% four, 2.3% five, and 1.2% six. Therefore, this assay will be very important to assess associations between bacteria in the vaginal microbiota.

Individual analysis of each of the BV-AAs revealed that the most frequently detected BV-AA as a single or simultaneous agent was* G. vaginalis* (*n* = 74; 45.7%). Historically,* G. vaginalis *was thought to play the leading role in infection, which created a niche suitable for colonization by strict anaerobes, which are largely responsible for the clinical symptoms of BV [49–51]. Recent published findings suggested that* G. vaginalis* biofilms may be critical in BV pathogenesis and symptomatology [24]. However, the detection of only one bacteria type is not a specific marker of BV because it may be commonly present in women with normal vaginal flora, although generally in smaller numbers than in women with BV [6, 27, 52].

*Megasphaera* type I was the second most frequent BV-AA (32.1%), and the third most frequent was BVAB 1 (*n* = 35; 21.60%).* M.* type I is an anaerobic bacterium similar to lactobacilli that produces lactic acid, which is strongly related to BV and correlated significantly with increased sexual exposure [27]. Fredricks et al. [34] reported that these bacteria are excellent markers of BV either alone or in combination with other BV-AAs. Fethers et al. [27] showed that the detection of the combination of either *M*. type I or one of the* Clostridiales* bacteria (BVAB 1–3) using PCR yielded a sensitivity of 99% and a specificity of 89% for the diagnosis of BV [27, 34]. The other BVABs (2 and 3) were detected in the present study at a lower frequency than BVAB 1 (12.3% and 16.7% of samples, resp.). A previous study showed that the detection of BVAB (1–3) was highly related to the presence of BV. The presence of BVAB (1–3) resulted in BV a few months after analysis, despite the detection of these bacteria in the vaginal fluid of healthy women [32]. Therefore, our results for the detection of *M*. type I, BVAB 1, BVAB 2, and BVAB 3 supports their importance in BV physiopathology and further demonstrated that our technique has great reliability and potential use for BV screening.

*U*.* urealyticum* and* M. curtisii* were detected with intermediate frequency (17.3%, and 13.6%, resp.), and other BV-AAs were detected much lesser frequently:* A. vaginae* (9.2%),* M. hominis, *and* S. sanguinegens* (5.5%, each),* M. mulieris* (3.7%),* B. fragilis *(2.5%), and* M. genitalium* (1.2%). Different molecular studies do not unanimously identify BV markers with respect to frequencies and the bacterial agents detected. Some findings are more common, but there is a wide variation [26, 27, 29]. For example, Pépin et al. [26] reported that the presence of* G. vaginalis*,* Bifidobacterium*,* Megasphaera elsdenii*,* Dialister*,* M. hominis*,* Leptotrichia*, and* Prevotella* was independently associated with BV. However,* Mobiluncus*,* A. vaginae*,* Anaerococcus*, and* Eggerthella* were not independently associated with BV. Fethers et al. [27] reported that only* M*. type I, BVAB 2,* A. vaginae,* and* G. vaginalis* were significantly associated with BV. Shipitsyna et al. [29] showed that only* G. vaginalis*,* A. vaginae*,* Eggerthella*,* Prevotella*, BVAB 2, and *M*. type 1 were highly predictable for BV. Twin et al. [53] detected most bacteria of the genus* Prevotella* (predominately* P. amnii*), followed by* Megasphaera*,* Leptotrichia*/*Sneathia,* and* Fusobacterium* (8%), of which* P. amnii* was strongly associated with BV. These variations between studies may be influenced by the type of women studied (e.g., pregnant or not; with or without symptoms of BV; with differences in the frequency and type of sexual contact, etc.) or the type of molecular technique used. Therefore, we chose a roster of BV-AAs that are commonly found in women with BV in previous studies for inclusion in our M-PCR assay.

Our M-PCR assays are qualitative and do not provide information about the quantities of bacteria that are present in subjects with and without BV. The quantity of bacteria may be an important predictor of disease [34]. However, we proposed a technique that is more economically accessible and easier to use as screening tool to benefit women with a molecular diagnosis, even in regions and/or countries with less financial resources. Additionally, we did not attempt to assay every known vaginal bacterium using targeted PCR assays because of practical limitations. We expect that many additional bacterial species are present in vaginal samples from subjects with and without BV. Nevertheless, these results build a foundation to improve our understanding of bacterial diversity in the human vagina.

Our data support that the detection of thirteen BV-AAs using M-PCR provides important information about the frequency of these agents. Additionally, M-PCR should be applied for the diagnosis or confirmation of BV, which would lead to an earlier diagnosis to prevent possible complications in specific women, without the impediments of high cost, long assay times, and difficulties in workflow. Finally, M-PCR provided information to improve our understanding of this syndrome, which may improve the management and optimal medical treatment for women with BV.

## Figures and Tables

**Table 1:** Nucleotide sequences of amplification primers used in the M-PCR.

BV-AAs/primers	Sequence (5′-3′)	Amplicon size (bp)	Reference or source^∗^
M-PCR assay 1			
*Mobiluncus curtisii *			[37]
Forward Reverse	GCCAGCCTTCGGGGTGGTGT TCACGAGTCCCCGGCCGAACC	130	
*Ureaplasma urealyticum *			[38]
Forward Reverse	AGAAGACGTTTAGCTAGAGG ACGACGTCCATAAGCAACT	541	
*Mycoplasma hominis *			[39]
Forward Reverse	ATACATCGATGTCGAGCGAG CATCTTTTAGTGGCGCCTTAC	270	
*Gardnerella vaginalis *			[40]
Forward Reverse	TTACTGGTGTATCACTGTAA CCGTCACAGGCTGAACAGT	330	
*Megasphaera *type 1			[41]
Forward Reverse	GATGCCAACAGTATCCGTCCG CCTCTCCGACACTCAAGTTCGA	211	
*Bacteroides fragilis *			[42]
Forward Reverse	TTCGCTTTTCTGTTTTCTGTGT CAGCAACCACCCAAACATTATT	842	

M-PCR assay 2			
*Atopobium vaginae *			[43]
Forward Reverse	TAGGTCAGGAGTTAAATCTG TCATGGCCCAGAAGACCGCC	155	
BVAB1			[41]
Forward Reverse	GGAGTGTAGGCGGCACTA CTCTCCGATACTCCAGCTCTA	90	
BVAB2			[41]
Forward Reverse	TTAACCTTGGGGTTCATTACAA GAATACTTATTGTGTTAACTGCGC	260	

M-PCR assay 3			
BVAB3			[41]
Forward Reverse	CATTTAGTTGGGCACTCAGGC ACATTTGGGGATTTGCTTCGCC	160	
*Mycoplasma genitalium *			[44]
Forward Reverse	ACCTTGATGGTCAGCAAAACTT CCTTTGATCTCATTCCAATCAGTA	193	
*Mobiluncus mulieris *			[24]
Forward Reverse	ATGGATATGCGTGTGGATGG CCAGGCATGTAAGCCCAAA	80	
*Sneathia sanguinegens *			[41]
Forward Reverse	AATTATTGGGCTTAAAGGGCATC AGTACTCTAGTTATACAGTTTTGTAG	102	

M-PCR: multiplex polymerase chain reaction; BV-AAs: bacterial vaginosis-associated agents; bp: base pairs; BVABs 1, 2, and 3, bacterial vaginosis-associated bacteria 1, 2, and 3.

^∗^Published primers were used without modification for all bacteria but were previously checked against all sequences in GenBank and evaluated by performing a BLAST analysis.

**Table 2:** M-PCR validation based compared to sPCR for thirteen major BV-AAs in cervical-vaginal samples.

Agents	Sensibility (%)	Specificity (%)	PPV (%)	NPV (%)	Accuracy (%)
**Overall**	99.1	100.0	100.0	97.0	99.3
*Mobiluncus curtisii *	100.0	100.0	100.0	100.0	100.0
*Ureaplasma urealyticum *	100.0	100.0	100.0	100.0	100.0
*Mycoplasma hominis *	80.0	100.0	100.0	97.8	98.0
*Gardnerella vaginalis *	100.0	100.0	100.0	100.0	100.0
*Megasphaera *type 1	100.0	100.0	100.0	100.0	100.0
*Bacteroides fragilis *	100.0	100.0	100.0	100.0	100.0
*Atopobium vaginae *	100.0	100.0	100.0	100.0	100.0
BVAB1	100.0	100.0	100.0	100.0	100.0
BVAB2	88.8	100.0	100.0	97.6	98.0
BVAB3	100.0	100.0	100.0	100.0	100.0
*Mycoplasma genitalium *	100.0	100.0	100.0	100.0	100.0
*Mobiluncus mulieris *	100.0	100.0	100.0	100.0	100.0
*Sneathia sanguinegens *	100.0	100.0	100.0	100.0	100.0
**Two or more agents simultaneously**	100.0	100.0	100.0	100.0	100.0

M-PCR: multiplex polymerase chain reaction; sPCR: single polymerase chain reaction; BV-AAs: bacterial vaginosis-associated agents; PPV: positive predictive value; NPV: negative predictive value.

**Table 3:** M-PCR assay performance in 45 initial samples analyzed from women with BV diagnosis by Nugent criteria.

BV-AAs detected	*n*	%
Only one BV-AA	**20 **	**44.5**
*Megasphaera* type I	9	45.0
*Mobiluncus curtisii *	6	30.0
*Gardnerella vaginalis *	4	20.0
*Mycoplasma hominis *	1	5.0
2 simultaneous BV-AAs	**10 **	**22.2**
*Ureaplasma urealyticum* + *Gardnerella vaginalis *	2	20.0
*Megasphaera* type I + BVAB 1	2	20.0
*Mobiluncus curtisii* + BVAB 1	1	10.0
*Megasphaera* type I + *M. hominis *	1	10.0
*Megasphaera* type I + *Atopobium vaginae *	1	10.0
*Mycoplasma hominis* + *Sneathia sanguinegens *	1	10.0
*Gardnerella vaginalis* + BVAB 3	1	10.0
*Mobiluncus curtisii* + BVAB 2	1	10.0
3 simultaneous BV-AAs	**6**	**13.3**
*Megasphaera* type I + BVAB 1 + BVAB 3	2	33.3
*Gardnerella vaginalis* + BVAB 2 + BVAB 3	1	16.6
*Mobiluncus curtisii* + BVAB 1 + BVAB 3	1	16.6
*Megasphaera* type I + BVAB 2 + BVAB 3	1	16.6
*Gardnerella vaginalis* + *Atopobium vaginae* + BVAB 2	1	16.6
4 simultaneous BV-AAs	**6**	**13.3**
*Megasphaera* type I + *Atopobium vaginae* + BVAB 2 + *Sneathia sanguinegens *	1	16.6
*Gardnerella vaginalis* + *Mobiluncus curtisii* + BVAB 2 + *Sneathia sanguinegens *	1	16.6
*Gardnerella vaginalis* + *Mobiluncus curtisii* + BVAB 2 + *Atopobium vaginae *	1	16.6
*Gardnerella vaginalis* + *Atopobium vaginae * + BVAB 2 + BVAB 3	1	16.6
*Megasphaera* type I + *Atopobium vaginae* + BVAB 2 + BVAB 3	1	16.6
*Megasphaera* type I + BVAB 3 + *Mobiluncus mulieris* + *Sneathia sanguinegens *	1	16.6
5 simultaneous BV-AAs	**2**	** 4.4**
*Ureaplasma urealyticum* + *Gardnerella vaginalis* + *Atopobium vaginae* + BVAB 1 + BVAB 2	1	50.0
*Mobiluncus curtisii* + *Megasphaera* type I + BVAB 3 + *Mobiluncus mulieris* + *Sneathia sanguinegens *	1	50.0
6 simultaneous BV-AAs	**1**	** 2.2**
*Gardnerella vaginalis* + *Mobiluncus curtisii* + *Megasphaera* type I + *Atopobium vaginae* + BVAB 2 + *Mobiluncus mulieris *	1	100.0

M-PCR: multiplex polymerase chain reaction; BV: bacterial vaginosis; BV-AAs: bacterial vaginosis-associated agents; BVABs 1, 2, and 3, bacterial vaginosis-associated bacteria 1, 2, and 3.
